# Boston Dental College

**Published:** 1899-07

**Authors:** 


### EDITORIAL.

### Boston Dental College.

From information recently received it appears that the Boston Dental College has undergone a great change. It has, as we understand, been transferred to and will become a department of Tuft's College. It seems that the old board of trustees will have no part in the matter in the future. In this change all the property belonging to the college has passed into the hands of Tuft's College management. We are not advised as to whether all or any considerable portion of the old faculty will be connected with the new department of Tuft's. It is to be hoped that whoever may have charge of its management will see that it is made a first-class institution. If this is attained, it will have the best wishes of all truly professional dentists in the country. If, however, there is a failure in this respect, it would be better had it not started on a new career. We will hope the best things for it, however.

